# Interesting Cases in Private Practice

**Published:** 1866-11

**Authors:** Andrew J. Scott

**Affiliations:** Londonville, Ohio


ART. II.—*Interesting Cases in Private Practice.* BY ANDREW J. SCOTT, M. D.

There is much in our daily practice that would not bear rehearsal, because of such frequent occurrence, and sameness in both symptoms and treatment. But with any of us there will occasionally a case make its appearance, worthy of more than a passing notice; a case indeed extraordinary in character, differing widely from those ordinarily falling under our care and observation. It is to exhibit a few of these cases, as I find them recorded in my daily memoranda, that I write, and not with the intention of attempting an elaborate essay on any disease or set of symptoms, or to set forth anything different from what may have been seen or held to by other members of the profession, and with the additional incentive of seeing myself as others see me, hoping I may be enabled thereby to correct errors in my practice.

Another inducement to write is, the fact that while we have a fair supply of reports of cases treated in hospital practice, we find but a meagre display of private cases, or those treated in what is sometimes termed the humbler spheres of the profession. The marked case in private practice may possess an equal and perhaps a greater interest to the general practitioner than one reported from the ward of a hospital, because even physicians are to some extent creatures of circumstance, and the responsibilities of the private practitioner are probably greater than those assumed in a hospital; as in the latter he is responsible only to himself, his patient, and the dispenser of events, while in the former position in addition to all these he has the weight of public opinion to exercise its influence over his actions, or at least over his mental cogitations, as he sits alone looking over the past; and if there is a power behind the throne that is even greater than the throne itself, that power is public opinion.

Typhoid fever, or as Prof. Wood has properly termed it, enteric fever, made its first appearance here in autumn of 1845, and from the account given by our older physicians, prevailed to an alarming extent, and was very fatal in character. It has prevailed to some extent here at some time during every year since, sometimes sporadically, and at other times affecting many persons in a given district, giving the most positive evidence of its contagiousness, and not apparently influenced by physical causes; prevailing alike on the table lands and the low, mashy districts. This disease presented all the well marked enteric symptoms as portrayed by the distinguished author above mentioned, so much so indeed that the glandular lesion of the intestinal canal was always sought for and readily recognized when found.

During the year 1864, and a part, if not all, of 1865, although we had fully the usual amount of febrile disease, most of which was typhoid in character, the peculiar lesion affecting the glands of the colon, was seldom or never present, and the prevailing fever justly entitled to be called "typho-malarial." This type of fever had prevailed here during that period so generally that we had almost ceased to expect a case of well marked enteric fever.—However, during the last of 1865 or first part of 1866, enteric fever again made its appearance, and was well marked in character, though it did not prevail to a great extent, and usually resulted in recovery under ordinary good treatment. As winter merged into spring, instead of this disease losing its severity with the season's change, and growing more mild, it unfortunately took on a complication both unusual and difficult to manage; a complication different indeed from anything that had fallen under my observation and consisting of cases of well marked enteric fever complicated with severe and in some cases fatal rheumatism.

I had (as I believe in common with most of the profession) embraced the doctrine that rheumatism was owing in a great degree to the presence of certain acids in the blood, thus pervading the whole system, and that this pathological condition was properly combated by alkalies; and that in enteric fever there already existed an alkaline state of the blood indicating the two diseases as diametrically opposed to each other, and incompatible. But whether in accordance with previously received theories or not, or even overthrowing the same, certain it was that these two diseases did affect the same individual at the same time, as indicated by the most positive and well marked symptoms belonging to each. How this could be is a physical phenomenon I shall not attempt at present to explain, simply wishing to present the facts as manifested in the few cases that fell under my immediate observation. My observation extended to five cases, with brief sketches of some cases that were in charge of neighboring physicians. Of these cases none had convalescence fairly established under twenty days, some not earlier than the thirtieth day, one of which proved fatal. The treatment adopted was about the same in all the cases, and may have been proper in all, and consisted principally of anodynes and nourishment with occasional doses of alkalies and arterial sedatives.

As the cases presented a marked sameness and were treated almost identically, the report of a single case is that of all, except that of the fatal case during the last four days of his illness; and this change of treatment unfortunately produced no apparent effect. Referring to my memoranda I find case hastily sketched as follows:

On the evening of April 12th, was called to see M. F., a bright boy of 13 years, and learned that he had first been taken ill on the 10th, when his mother had administered a mild cathartic, as she supposed, but which had proved very active, continuing to disturb his bowels up to the time when I was called; found his pulse 100 per minute and small; face flushed, tongue diminished in volume, dry, coated and red at the point; bowels tender and tympanic, with marked regurgitation in the region of the colon; pain in the head, limbs and back, with a general uneasiness for which he could not give any particular reason. Gave Dover's powder, gr. vi, every four hours, with fld. ext. veratrum viride, gt. v, half way between each two powders; mustard and warm applications over his bowels, cold to his head and occasional spongings with tepid water.

13. Patient much as when last seen. Added pulv. opii gr. ss to the powders, and continued treatment.

14. Pulse 90; had rested better; had taken more nourishment, and had not had any stool. Continued treatment.

15. Pulse 90; rested some since last seen; bowels quiet and less tympanitic, but complained of severe pain in his right hand, which was red and swollen. Ordered Rochell salts, to be followed, after operation, by powd. opii gr. ii. every five hours, and fl. ext. verat. with iod. potass. gr. x. in solution between the opium powders.

18. Pulse 80; had rested well during the night; tongue cleaning off, somewhat moist, with an improved condition of the bowels, rendering his case to all appearances hopeful, though his hand was still some swollen and slightly painful. Continued treatment, only giving the medicine at longer intervals.

17. Patient as when last seen; bowels had moved once. Continued treatment.

18. Doing well. Diminished the quantity of iod. potass. and continued other medicine.

19. Rested well since last seen; pulse 80; swelling of hand nearly disappeared, with every indication favorable. Gave pulv. opii. gr. ss. every six hours, with iod. potass. gr. v. every six hours.

20. Not so well; pulse 90; tongue very red, with a varnished appearance; bowels tender and tympanitic; had purged several times, discharging yellow ochery stools; right foot and ankle swollen and very painful. Increased the quantity of opium and iod. potass., and also gave turpentine emulsion.

21. Pulse 100; no other change. Continued treatment, and endeavored to control the circulation with veratrum.

22. No change. Continued treatment.

23. Pulse 120; both feet swollen and very painful; all the symptoms aggravated. Increased the quantity of opium, and continued treatment.

24. Pulse 130, subsultus; purging, notwithstanding the heavy portions of opium; feet swollen, very painful still. Continued opium and veratrum.

25. 8 A. M. Saw the patient in consultation with Dr. Robison, of Wooster. No change since yesterday, except, on examination, a distinct bellow's murmur of the heart was apparent, which, with the previous history of the case, but too plainly indicated that the rheumatism had invaded that organ. At the suggestion of Dr. R., (in whom I have much confidence,) we put the patient on sulphite soda, gr. xx. every four hours, with spirits nitre, saturated with acetate potass. between each two portions of the sulphite soda, with sufficient opium to secure rest.

26. Very little change. Continued treatment.

27. Patient failing; both the enteric and rheumatic symptoms aggravated in character. Continued treatment, though it did not promise relief, it being necessary to give large portions of sulph. morphia in order to secure any degree of quiet. Attempted to give stimulants in the afternoon, but they evidently increased the excitement and did harm—it being necessary to withdraw them.

28. Patient failing rapidly, and died at 5 P. M., after an illness of eighteen days.

Tenderness, regurgitation and tympanitis of the bowels or some part of the abdominal viscera were present all the time during his illness, with a constant disposition to purge unless prevented by the use of opium. I am fully satisfied that a post mortem examation (which I regretted could not be obtained) would have exhibited in the intestinal canal all the glandular disease present in uncomplicated cases of enteric fever, sufficient to have jeopardized the life of the patient.

The rheumatic disease, as manifested in the swollen and reddened joints, was constantly present in some degree after the fifth day of the disease; was only partially modified by the use of alkalies, which appeared quite powerless in the later stage of the case, though the urine was easily rendered strongly alkaline at any time. The immediate cause of death was rheumatism affecting the heart, a condition I had anticipated with fear, as seen in similar cases; had endeavored to prevent and finally to remove, but without success.

Of the use of sulphite of soda, which has been lauded for such cases, I am unable to express an opinion, as this was the only case in which I used it—at a late stage, and what appeared to be doomed a fatal case. Nothing appeared to produce any lasting benefit, except some form of opium, which calmed the troubled frame and allowed the spirit to take its flight with less of suffering. This was the last case of that kind in this vicinity, our community having enjoyed an unusual immunity from all febrile diseases during the summer just past, and only a few cases, and they generally of a mild type, making their appearance at the present time.

LONDONVILLE, OHIO, November, 1866.

